# Articulatory Changes in Vowel Production following STN DBS and Levodopa Intake in Parkinson's Disease

**DOI:** 10.1155/2015/382320

**Published:** 2015-10-07

**Authors:** Vincent Martel Sauvageau, Johanna-Pascale Roy, Léo Cantin, Michel Prud'Homme, Mélanie Langlois, Joël Macoir

**Affiliations:** ^1^Centre Interdisciplinaire de Recherche en Réadaptation et en Intégration Sociale, Québec, QC, Canada G1M 2S8; ^2^Centre Interdisciplinaire de Recherche sur les Activités Langagières, Université Laval, Québec, QC, Canada G1V 0A6; ^3^Département des Sciences Neurologiques, CHU de Québec (Hôpital de l'Enfant-Jésus), Québec, QC, Canada G1J 1Z4; ^4^Centre de Recherche, Institut Universitaire en Santé Mentale de Québec, Laboratoire Langage et Cognition, Québec, QC, Canada G1J 2G3

## Abstract

*Purpose*. To investigate the impact of deep brain stimulation of the subthalamic nucleus (STN DBS) and levodopa intake on vowel articulation in dysarthric speakers with Parkinson's disease (PD). *Methods*. Vowel articulation was assessed in seven Quebec French speakers diagnosed with idiopathic PD who underwent STN DBS. Assessments were conducted on- and off-medication, first prior to surgery and then 1 year later. All recordings were made on-stimulation. Vowel articulation was measured using acoustic vowel space and formant centralization ratio. *Results*. Compared to the period before surgery, vowel articulation was reduced after surgery when patients were off-medication, while it was better on-medication. The impact of levodopa intake on vowel articulation changed with STN DBS: before surgery, levodopa impaired articulation, while it no longer had a negative effect after surgery. *Conclusions*. These results indicate that while STN DBS could lead to a direct deterioration in articulation, it may indirectly improve it by reducing the levodopa dose required to manage motor symptoms. These findings suggest that, with respect to speech production, STN DBS and levodopa intake cannot be investigated separately because the two are intrinsically linked. Along with motor symptoms, speech production should be considered when optimizing therapeutic management of patients with PD.

## 1. Introduction

Parkinson's disease (PD) is commonly viewed as a multisystemic degenerative disorder [1]. Alongside motor symptoms such as tremor, muscle rigidity, and bradykinesia, up to 90% of people with PD develop speech disorders over the course of the disease [2]. Speech impairment in PD has been studied in many investigations from a motor, acoustic, or perceptual point of view. Studies examining physiological changes in the speech systems of people with PD reported altered respiratory [3], laryngeal [4], and orofacial [5, 6] functions. These motor manifestations have impacts on the acoustic signal of speech, such as reduced intensity level [7] and fundamental frequency (*f*_0_) range [8], altered phonation quality [9], and inaccurate and reduced articulation [10–12]. All these changes affect listeners' perceptions, such as perceived softer speech, reduced voice quality, and poor articulation. As a result, impaired intelligibility is very common in PD [13]. These motor, acoustic, and perceptual changes are grouped under the term “hypokinetic dysarthria” [14].

Multiple pharmacological and surgical techniques are now available to help manage the different motor symptoms of patients with PD. For all patients, pharmacotherapy with levodopa remains the focal point of therapeutic management. However, over the years levodopa use may induce side effects such as dyskinesia and dystonia, and these adverse effects tend to increase with the dose. The impact on speech production is still not fully understood but is generally mixed or poor. For example, Goberman et al. [15] reported that levodopa intake may slightly improve phonation quality in some patients. On the other hand, other papers such as De Letter et al. [16] reported that it could lead to altered speech rate. For a review of the impact of levodopa intake on speech production in PD, see Schulz and Grant [17].

Different surgical techniques have been developed in recent decades to complement pharmacological treatments in people with PD. One of these surgical techniques is deep brain stimulation of the subthalamic nucleus (STN DBS). The impact of STN DBS on various symptoms in PD can be investigated using two different research paradigms: (1) measuring the changes that occur directly with electrical stimulation (on-/off-stimulation designs) or (2) measuring the longitudinal changes that occur with the surgery itself (pre/postsurgery designs). The latter approach is interesting because it involves documenting the impact of both the electrical stimulation and the surgery itself. Even though it has been demonstrated that STN DBS can drastically reduce motor symptoms and improve patients' quality of life [18], it has been associated with relatively small changes in dysarthria severity levels and intelligibility. Most on-/off-stimulation studies of speech production showed that the impact electrical stimulation has is at best mixed and more often than not minor and/or poor. For a review, see Skodda [19]. Pre/postsurgery studies also found mixed results. Pinto et al. [20] reported improvement in some aspects of speech at 1 to 5 years after surgery, while Wang et al. [21] reported altered respiration and phonation 3 months after surgery. They hypothesized that this deterioration could be explained by the microlesions that occur during the surgical procedure itself.

One of the targets of STN DBS is to functionally replace some of the anti-Parkinson drugs used to manage motor symptoms in PD. Most patients need lower levodopa doses following STN DBS. Consequently, both STN DBS and levodopa intake must be taken into account when investigating changes that occur with therapeutic management.

Speech production comprises different specific components, one of which is articulation [22]. The articulation of speech sounds requires fine motor control. Speech units can be characterized in terms of articulatory gestures (range of movement), or in terms of acoustic distinctiveness. This second approach assumes that two speech units that are acoustically differentiated are more easily identified by our perceptual system [23]. Using this paradigm, vowels can be described and differentiated from one another by their acoustic characteristics, namely, their formants. The first two formants of a vowel,* F*1 and* F*2, are spectral values that allow categorization of the phoneme.* F*1 and* F*2, respectively, serve as indicators of the open-close and front-back position of the articulators (jaw and tongue) during the production of speech [24]. The articulation of vowels is very important for speech intelligibility, and reduced acoustic distinctiveness of vowels has been reported in studies of dysarthric speakers, including people with PD [25]. Skodda et al. [26] reported that vowel articulation in this population did not vary with levodopa intake at the group level but that individual data for some participants showed improvements. In only a few studies did researchers investigate the changes STN DBS induces in vowel production. However, the objective of these studies was not articulation per se, but voice quality [27] or speech rate [28].

Recently, Tripoliti and colleagues [29] investigated the impact of STN DBS and levodopa intake on different components of speech intelligibility in PD in a cohort of 54 consecutive patients. One of the components they analysed was general articulation. Perceptual measures of articulation were completed by merging different factors of articulation (such as phoneme imprecision, prolongation, or repetition) into a global speech sign-cluster of articulation (see [30] for specific details regarding this clustering). They found that STN DBS could lead to increased perceived articulation impairment and that levodopa, on the other hand, could improve it. However, due to the nature of their study, no fine acoustic measurements of articulation were completed.

To our knowledge, no studies looked at the pre-/post-impact of STN DBS and levodopa intake, as well as the interaction between these two variables, on vowel articulation in PD, looking at their acoustic distinctiveness. The primary objective of this study was to examine the long-term effects of STN DBS on speech articulation in dysarthric speakers with PD, using acoustic measurements of vowel distinctiveness. The second objective was to explore the effect of levodopa intake on vowel articulation. The last objective was to examine if the change in levodopa intake following STN DBS might also modulate vowel articulation.

## 2. Method

### 2.1. Participants

#### 2.1.1. Demographic Characteristics

The study was approved by the local institutional ethics committee for the safety of human subjects and written informed consent was obtained from all participants. Seven consecutive participants (6 men and 1 woman, 65.9 ± 5.1 years) with idiopathic PD diagnosed 11.1 ± 2.8 years prior to the study were recruited in an outpatient clinic. All participants had already undergone the evaluation process for STN DBS and were accepted and eligible for surgery. All of them were native speakers of Quebec French who had always lived in the province of Quebec. Although no formal hearing evaluation was conducted, all participants were functional in conversation and none reported any hearing impairment. General cognitive functions were measured using the Montreal Cognitive Assessment (MoCA) [31] and no participant fell below the cut-off score according to age and education level [32]. Although the presence of a speech disorder was not a criterion to be eligible for this study, all participants reported speech difficulties. Their main complaints were (1) harsh and/or soft voice, (2) speech rate control difficulties, and (3) impaired articulation. These alterations were largely confirmed by the SLP that conducted the assessments. They are consistent with the speech impairments typically observed in hypokinetic dysarthria [13]. Even though quantitative measurement of speech intelligibility was not performed, dysarthria severity, as clinically reported by the SLP, ranged from mild to severe.

#### 2.1.2. Surgical Procedure and Deep Brain Stimulation Characteristics

The participants in this study underwent bilateral DBS of the STN done by the same neurosurgeons (LC and MP). Surgery was done under local anaesthesia and sedation with the CRW stereotactic frame. The day before surgery, patients had high-resolution T2-weighted MRIs (3.0-T unit, Siemens). These images were fused with a T1-Gadolinium (double dose, 1.5-T unit, Siemens) acquired with the localization frame on the day of surgery. Neuronavigation (Stealth System from Medtronic, Inc., Minneapolis, MN) was used to plan the surgery and fuse the images. The target was at the STN and it was calculated from the midcommissural point. The indirect coordinates were 3 mm behind the midcommissural point, 12 mm lateral, and 4 mm inferior. The target was confirmed by microrecording and microstimulation; then a quadripolar lead was implanted (Model 3387, Medtronic, Inc., Minneapolis, MN). Surgery was done on both sides the same day. One to 3 days later, the neurostimulator was implanted (Activa System, Medtronic, Inc., Minneapolis, MN).

After the surgery, the participants were regularly followed by the same neurologist (ML), who selected and adjusted the configuration of electrical parameters for each of them, based on observed and reported clinical symptoms of PD such as tremor, rigidity, speech difficulty, and dyskinesia. Anti-Parkinson drugs were also adjusted in parallel. Individual DBS parameters at one-year followup and levodopa daily dose before and after surgery for each participant are reported in Table 1.

### 2.2. Evaluation Sessions

Changes in vowel articulation were measured before/after STN DBS (the day before surgery; then 1 year later) and off-/on-medication (12 hours without medication; then 1 hour after taking it). Evaluations were thus done under 4 clinical conditions: before surgery in the off-medication state (Pre-op, Off-med), before surgery in the on-medication state (Pre-op, On-med), after surgery in the off-medication state (Post-op, Off-med), and after surgery in the on-medication state (Post-op, On-med). Both evaluations performed after the surgery were done with the internal pulse generator turned on (“on-stimulation”). No changes in the electrical stimulation parameters were made for at least three months prior to the evaluations.

#### 2.2.1. Neurological Assessment

At each evaluation session, the severity of motor symptoms was measured using the motor section of the Unified Parkinson's Disease Rating Scale (UPDRS-III). This neurological assessment was done for each participant to document the impact of levodopa as well as the long-term effects of STN DBS on motor symptoms.

#### 2.2.2. Speech Assessment

At each evaluation session, speech recordings were made at the hospital in a quiet room using a Shure 510A head-mounted microphone and a Zoom H4n audio recorder at a sampling rate of 44.1 kHz. Mouth-to-microphone distance was approximately 4 to 8 cm for each participant but remained constant throughout the same session. Vowel articulation was measured with a reading aloud task of spoken /pVpy/ tokens, with the vowels /i/ and /u/ and /a/ (e.g., “pipu” or “papu”) as targets. Even though French and English have different vocalic systems, the vowels /i/ and /u/ and /a/ occupy similar cardinal positions in both languages. Therefore, it was not expected that results would be language-dependent. The vowels were placed in this phonetic context to standardize coarticulation, that is, the impact speech units have on each other. These tokens were embedded in the carrier phrase “*V comme /pVpy/*” (e.g., “*A comme papu*”) in order to standardize the prosody and accentuation of productions. Each individual token was repeated 5 times, with a total of 15 productions per participant per recording session. The order of presentation of the tokens was randomized but this sequence remained the same across all participants and throughout all recording sessions.

### 2.3. Acoustic Analyses

The initial data pool of analyzed vowels contained 420 productions (3 vowels × 5 repetitions × 2 surgery states × 2 medication states × 7 participants). Due to the recording conditions and participants' fluctuating voice quality (e.g., oversaturation of the microphone, voice breaks, and no discernible glottal pulse), 14 data points (2.5%) could not be analyzed. Overall, 406 valid vowels were analyzed and comprised the final data pool. All acoustic analyses were done by a trained phonetician using Praat software v5.3.30 [33] running on Windows OS. Acoustic segmentations were conducted using different visual criteria on a spectrogram and oscillogram. Multiple scripting procedures were implemented in the analyses when no manual intervention was required.

Vowel articulation was measured by analyzing* F*1 and* F*2 formant frequencies of /i/ and /u/ and /a/ taken on the 20 ms midpoint in 500 ms analysis windows. Vowel duration was also measured for covariance analyses. Vowel onset and offset were first determined by the appearance of the first and last glottal pulse visible on the oscillogram. With these values, two variables were calculated. The first variable is the* acoustic vowel space* (AVS), which is the surface of the triangle formed by the* F*1 and* F*2 formant values of the vowels /i/ and /u/ and /a/. Higher AVS values correspond to* increased* vowel articulation.

The second variable is the* formant centralization ratio* (FCR), which is a coefficient that represents the magnitude of centralization of the formants* F*1 and* F*2 for vowels /i/ and /u/ and /a/. This metric was developed by Sapir et al. [34] and has been used in other studies on vowel articulation in PD. Higher FCR values represent higher formant centralization and consequently* reduced* vowel articulation. For a detailed description of the formulas used to calculate AVS and FRC, see [35].

### 2.4. Statistical Analyses

The effects of STN DBS surgery (Pre versus Post) and medication state (On versus Off) on each variable in the study were analyzed using a mixed model analysis of variance (ANOVA). Participants, vowel repetition, and the intercept for each participant were entered in the model as random factors and were based on a scaled identity covariance matrix. All dependent variables were analyzed using the surgery (Pre versus Post), medication (Off versus On), and their interaction (surgery*∗*medication) as fixed factors. When applicable, post hoc analyses (pairwise comparisons using Bonferroni correction) were done in order to determine statistical differences between recording sessions. For these analyses, the same random factors were entered but the order of the recording session was entered in the model as a fixed factor. All statistical analyses were done using* SPSS* v.20 [36]. Using this type of statistical analysis is generally the norm in phonetic research for two main reasons. First, it allows the model to take into account the normal variability of speech production in the population due to anatomical differences in the vocal tract shape. Second, it helps to avoid statistical problems like pseudoreplication of data associated with repetition of the same tokens multiple times. This procedure follows the guidelines suggested for phonetic research by Max and Onghena [37].

## 3. Results

### 3.1. Neurological Assessment

Initial analyses focused on levodopa equivalent dose in order to describe the changes that occurred following the surgery. There was a significant difference (*t*(6) = 5.22, *p* < 0.01) in the dose between Pre-op (M = 1273.7, SD = 353.1) and Post-op (M = 492.9, SD = 240.5).

Regarding UPDRS-III scores, statistical analyses indicate a significant effect of the surgery state: (*F*(1; 26.72) = 12.370, *p* < 0.01) Pre-op (M = 32.2, SD = 2.7) versus Post-op (M = 20.8, SD = 2.5), as well as the medication state: (*F*(1; 26.72) = 14.690, *p* < 0.001) On-med (M = 20.6, SD = 2.6) versus Off-med (M = 32.4, SD = 2.6). These results show that, independently of each other, STN DBS and medication help reduce motor symptoms in participants. A significant interaction effect of Surgery × Medication was also found:* F*(1; 10.80) = 7.735, *p* < 0.05, which indicates that the effect of medication on the motor symptoms was higher at Pre-op (−18.1) than at Post-op (−5.4).

### 3.2. Vowel Articulation

Table 2 reports means and standard deviations for vowel duration (msec.),* F*1/*F*2 AVS (Hz^2^), and FCR Pre-op and Post-op in both On-med and Off-med conditions. No significant change was found in vowel duration between any conditions (*p* > 0.05). This suggests that neither STN DBS nor levodopa intake influenced vowel duration. Regarding AVS (higher value = greater articulation), statistical analyses did not show any significant effect of overall STN DBS (*F*(1; 23.66) = 0.05, *p* > 0.05) or levodopa intake (*F*(1; 23.66) = 0.06, *p* > 0.05), but a significant interaction effect of both factors (Surgery × Medication) was found:* F*(1; 8.70) = 5.15, *p* < 0.05. This indicates that the impact of levodopa intake on the size of acoustic vowel space is modulated by STN DBS. Post hoc analyses were done but failed to demonstrate specific differences between any conditions.

Regarding FCR (higher value = poorer articulation), similar results were obtained. Statistical analyses did not show any significant effect of overall STN DBS (*F*(1; 24.27) = 0.01, *p* > 0.05) or levodopa intake (*F*(1; 24.27) = 0.60, *p* > 0.05), but a significant interaction effect (Surgery × Medication) was found:* F*(1; 9.28) = 13.91, *p* < 0.01. Post hoc analyses were done to identify specific differences between conditions. A statistical difference was found between the following:

(1) Off-med and On-med conditions Pre-op (*p* < 0.01), with higher FCR values On-med: Cohen's effect size value (*d* = 0.97) suggests a high practical significance;
(2) Pre-op and Post-op conditions Off-med (*p* < 0.05), with higher FCR values Post-op: Cohen's effect size value (*d* = 0.63) suggests a medium to high practical significance;
(3) Pre-op and Post-op conditions On-med (*p* < 0.05), with lower FCR values Post-op: Cohen's effect size value (*d* = 0.73) suggests a medium to high practical significance.

**Figure 1:**
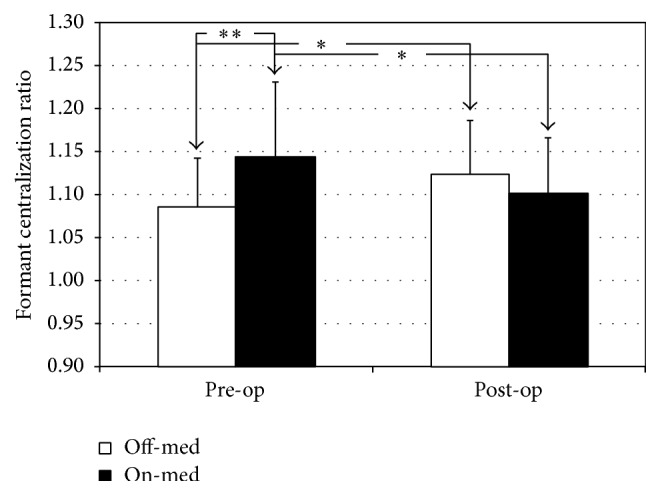
Formant centralization ratio (higher values = reduced articulation) before (Pre-op) and after (Post-op) STN DBS, off- and on-medication.

These results indicate that articulation range is (1) reduced with levodopa Pre-op; (2) reduced with STN DBS without levodopa intake; and (3) increased with STN DBS with levodopa intake. It is also to be noted that, on an individual level, each participant followed this trend. The group results are shown in Figure 1.

## 4. Discussion

This study reports results regarding changes in vowel articulation following bilateral STN DBS and levodopa intake in 7 individuals with PD. In order to do so, both treatments, as well as the interaction between them, must be examined specifically. Before undergoing STN DBS, medication significantly reduced vowel articulation in the patients reported in this study. Previous studies on the impact of levodopa intake reported that articulation was better with levodopa in some patients [26, 38]. However, our participants were about to undergo STN DBS, a treatment generally offered to patients who are less responsive to levodopa or who suffer from major side effects of the medication due to higher doses. In such patients, our results suggest that vowel articulation impairment is associated with high levodopa dose and that articulation impairment could even be a side effect of high levodopa use. On the other hand, one year postoperatively, the levodopa equivalent dose was significantly reduced because of the beneficial effects of the electrical stimulation on motor symptoms. This could explain why, at that time, levodopa intake no longer induced changes in vowel articulation. One possible explanation for this change in response to levodopa could be linked to the presence of facial or lingual dyskinesia. Prior to surgery, most patients showed signs of levodopa-induced dyskinesia, which diminished greatly in frequency and amplitude one year after surgery. However, no objective measures of the presence and severity of dyskinesias were taken in this study. This hypothesis should therefore be investigated more closely in future studies.

To examine the direct long-term effect of STN DBS on vowel articulation, Pre-op versus Post-op results must be compared only off-medication. Our results indicate that in this condition STN DBS induced articulatory impairment one year after surgery. Some previous studies reported deterioration in different speech components following STN DBS. Wang et al. [21] suggested that this could be attributable to the microlesions that occur during the surgical procedure itself rather than to the electrical stimulations. This hypothesis is also consistent with our results. However, more studies are needed to dissociate the impact of the surgery from the impact of electrical stimulation on speech articulation in PD. Research examining the long-term effects of STN DBS (Pre-op versus Post-op), the direct impact of stimulations (On-stim versus Off-stim), and the impact of levodopa intake (On-med versus Off-med) could provide answers. Such a study was published in the past [39] but vowel articulation was not one of the aspects investigated.

In general, the acoustic results in the present study are similar to those reported by Tripoliti and colleagues [29] on the impact of STN DBS in PD on the intelligibility of articulation. However, our results regarding the impact of levodopa intake and its change with STN DBS follow an opposite pattern. In summary, levodopa intake preoperatively impaired the articulation of the participants in the present study, whereas it had a positive effect on articulation in Tripoliti et al. study. One possible explanation for these opposite results could be due to the difference in clinical presentation of the dysarthria in participants of the two studies. A recent study conducted by Tsuboi and colleagues [40] specifically investigated the impact of STN DBS on speech disorders in multiple dysarthria phenotypes. They demonstrated that different phenotypes of dysarthria (based on the dominant altered speech components) respond differently to the stimulation. It is hypothesised here that their findings could also be applied to levodopa intake.

Overall, our results show the effects of STN DBS and levodopa intake on vowel articulation interact, suggesting that these variables should be considered when studying speech changes associated with therapeutic management in PD. Without taking medication into account, our results suggest that STN DBS impairs vowel articulation. However, when examining participants on-medication, our results indicate that vowel articulation is generally better 1 year postoperatively, probably because their medication is reduced. Although its importance was already raised in past studies, the interaction between these two therapeutic interventions is not always considered in research protocols investigating the impact of STN DBS on different symptoms in PD.

### 4.1. Limitations of the Study

Some limitations must be taken into consideration when interpreting the findings of this study. First, this investigation involved only a small number of participants. Generalization of these results must therefore be viewed with caution because people with PD exhibit large inter- and intrasubject variability in speech disorder characteristics and their motor response to STN DBS varies greatly. Another limitation is that the recordings occurred only preoperatively and one year postoperatively. Additional intermediate or follow-up assessments could have provided useful information regarding disease progression or attenuation to DBS. Finally, in this study, the articulation measurements were taken only in a reading task. The naturalness of speech in such tasks is debatable and this is commonly recognized as a limitation in phonetic studies [10, 41]. However, this methodological choice is the best way to control the phonemic, syntactic, and prosodic contexts around the target sounds.

The acoustic analysis of speech based on formants is particularly interesting in clinical settings since information in the signal is relatively resistant to environmental sounds or background noise. Moreover, subsequent analysis of these formants can be done relatively fast by a trained clinician. Acoustic metrics other than those used in this study (AVS and FCR) are available to describe articulatory changes in vowel production. For example, Weismer et al. [42] suggested that the transition on* F*2 in diphthong (e.g., /ei/ in “hail”), measured by the magnitude of the* F*2 slope, yields promising results in characterizing intelligibility changes with STN DBS in people with PD. This measure could therefore complement the acoustic measurements of vowel articulation conducted in our study. Such phonetic targets also have the advantage of being real words, as opposed to the nonwords used here, and could reflect more natural speech.

This study looked only at acoustic changes in vowel articulation that occur following STN DBS as well as levodopa intake. The impact of these changes on speech intelligibility was not examined here. Although vowel articulation is strongly associated with intelligibility, future studies should look at the direct association between acoustic change in vowel production and perceptual measurements such as vowel goodness or overall speech intelligibility. Furthermore, a specific acoustic production can be achieved using different motor sequences [42]. The relationship between acoustic/motor productions should also be investigated in future studies using acoustic as well as kinematic measurements.

## 5. Conclusions

This study is one of the first to specifically investigate changes in vowel articulation that occur in postoperative STN DBS on- and off-levodopa. Without taking levodopa into account, we found that STN DBS altered vowel articulation. However, 1 year after surgery, the participants had reduced levodopa doses, which had a positive effect on their articulation, compared to the period before surgery, on-medication. Therefore, clinical presentation of speech articulation may improve with STN DBS but this could be due mainly to the change in medication following surgery. Thus, speech properties should be included in the symptoms of PD when optimizing medication for patients.

## Figures and Tables

**Table 1:** Individual levodopa daily dose before and after surgery and DBS parameters at one-year followup.

Patient	L-dopa equivalent (mg)		Frequency (Hz)		Voltage (V)		Pulse width (*µ*s)	
	Pre-op	Post-op	Left	Right	Left	Right	Left	Right
1	1500	300	130	130	2.8	3.6	60	60
2	1688	650	140	140	2.5	2.5	60	60
3	1200	750	140	140	2.9	2.9	60	60
4	850	600	140	140	2.0	3.8	60	60
5	1684	600	140	140	3.7	3.7	90	60
6	1094	50	140	140	3.0	3.0	60	60
7	900	500	130	130	1.2	3.3	60	60

**Table 2:** Means (standard deviations) for vowel duration (msec.), acoustic vowel space (Hz^2^), and formant centralization ratio before and after STN DBS, off- and on-medication.

	Pre-op		Post-op	
	Off-med	On-med	Off-med	On-med
Vowel duration (msec.)	105.0 (24.7)	103.2 (31.1)	102.6 (18.7)	102.8 (22.4)

Acoustic vowel space (Hz^2^)	246006 (48045)	222166 (73934)	222430 (45823)	236810 (69166)

Formant centralization ratio	1.09 (0.06)	1.14 (0.09)	1.12 (0.06)	1.10 (0.07)
